# Enhanced audio-tactile multisensory interaction in a peripersonal task after echolocation

**DOI:** 10.1007/s00221-019-05469-3

**Published:** 2019-01-07

**Authors:** Alessia Tonelli, Claudio Campus, Andrea Serino, Monica Gori

**Affiliations:** 1 U-VIP: Unit for Visually Impaired People, Istituto Italiano di Tecnologia, Genoa, Italy; 2 MySpace Lab, Department of Clinical Neurosciences, University Hospital Lausanne (CHUV), Lausanne, Switzerland; 3 Center for Neuroprosthetics, School of Life Sciences, Ecole Polytechnique Fédérale de Lausanne, Lausanne, Switzerland

**Keywords:** Multisensory, Echolocation, Peripersonal space, Audio-tactile

## Abstract

Peripersonal space (PPS) is created by a multisensory interaction between different sensory modalities and can be modified by experience. In this article, we investigated whether an auditory training, inside the peripersonal space area, can modify the PPS around the head in sighted participants. The auditory training was based on echolocation. We measured the participant’s reaction times to a tactile stimulation on the neck, while task-irrelevant looming auditory stimuli were presented. Sounds more strongly affect tactile processing when located within a limited distance from the body. We measured spatially dependent audio-tactile interaction as a proxy of PPS representation before and after an echolocation training. We found a significant speeding effect on tactile RTs after echolocation, specifically when sounds where around the location where the echolocation task was performed. This effect could not be attributed to a task repetition effect nor to a shift of spatial attention, as no changes of PPS were found in two control groups of participants, who performed the PPS task after either a break or a temporal auditory task (with stimuli located at the same position of echolocation task). These findings show that echolocation affects multisensory processing inside PPS representation, likely to better represent the space where external stimuli, have to be localized.

**Electronic supplementary material:**

The online version of this article (10.1007/s00221-019-05469-3) contains supplementary material, which is available to authorized users.

## Introduction

Space is a construction of our brain and mind. Several lines of evidence show that our brain continuously generates multiple neural representations of coexisting spaces, depending on incoming sensory inputs, action/intention, and reference frames (McNaughton and Nadel 1990; Holmes and Spence 2004; Pasqualotto et al. 2013). An interesting spatial representation, which is nowadays attracting a renewed interest, is the peripersonal space (PPS), i.e., the space immediately surrounding the body (Rizzolatti et al. 1997; Ladavas and Serino 2008; Cléry et al. 2015; Dijkerman and Farnè 2015; Serino 2016). Studies show that PPS is represented by the integration between somatosensory stimuli from the body and visual (Làdavas et al. 1998; Macaluso and Maravita 2010) or auditory stimuli (Occelli et al. 2011) from the environment, when they are presented at a limited distance from the body. This integration defines the extent of the PPS (Bassolino et al. 2015; De Vignemont and Iannetti 2015). Interestingly, PPS representation has a direct link to the motor system, as stimuli presented within the PPS prime defensive (Graziano and Cooke 2006) or approaching (Rizzolatti et al. 1997) body actions (Cardinali et al. 2009; Serino et al. 2009; Makin et al. 2009; Avenanti et al. 2012).

An important property of PPS representation is that it dynamically modifies through experience, i.e., by short (Farnè and Làdavas 2000; Holmes and Spence 2004; Holmes et al. 2004; Canzoneri et al. 2013b) and long-term (Serino et al. 2007) tool-use, social interaction (Heed et al. 2010; Ferri et al. 2013; Teneggi et al. 2013; Pellencin et al. 2018) and potential movements (Brozzoli et al. 2010; Noel et al. 2015).

In this study, we investigated whether a novel form of exploring and interacting with the environment through sounds (echolocation) shapes PPS representation. Echolocation is based on the ability to measure the time delay between a sound and any echoes reflected by the environment. Specifically, using self-generated sounds, expert echolocators are able to navigate and detect an object present in the environment (Supa et al. 1944; Kolarik et al. 2014). Therefore, echolocation can be conceived as a form of tool-use able to modify the PPS. Echolocation can be used to “reach” sectors of space which are normally out of reach without visual information, thanks to the interpretation of the echoes produced by sound reflections on the objects. Recent studies have demonstrated that also sighted people, after a training, are able to perform simply echolocation tasks, such as size discrimination (Teng 2011; Thaler et al. 2014), detection task (Schenkman and Nilsson 2010; Thaler and Castillo-Serrano 2016) or bypassing obstacles (Kolarik et al. 2016; Tonelli et al. 2018). In a recent study (Tonelli et al. 2016), we trained sighted people in a depth echolocation task. Afterward few hours of training, participants were able to estimate objects depth with good accuracy and precision.

In the present study, we adopted an echolocation training in healthy participants, and then tested whether such training can modify PPS. To this aim, we evaluated the PPS around the head before and after an echolocation detection task, in which participants had to detect the presence of an object inside the peripersonal space by self-generated mouth clicks. To quantify the PPS, we adopted a behavioral measure, extensively used in previous studies to assess PPS in humans (Canzoneri et al. 2012, 2013a; Teneggi et al. 2013; Finisguerra et al. 2015; Noel et al. 2015) and to investigate the effects of different experimental manipulation and training on PPS representation (Canzoneri et al. 2013a, b; Bassolino et al. 2015; Maister et al. 2015; Salomon et al. 2017). In this task, participants had to respond as fast as possible to a tactile stimulus applied to their body, while task-irrelevant sounds were presented, giving the impression of a looming sound. Previous results showed that sounds speeded up the detection of tactile stimuli specifically when presented at a certain distance from the participants (and not farther from them). Such distance can be measured as a proxy of the extent of the participant’s PPS (Serino et al. 2015a, b). In addition to the experimental group, the same PPS task was administered to two control groups of participants to directly link any change in PPS representation to echolocation and to exclude general effects of increasing attention for an auditory stimulus at a given spatial location or task repetition. Thus, one control group performed the PPS task before and after a perceptual training, involving mainly temporal components (auditory time bisection), and the other group took simply a break between the two sessions of the PPS task.

## Methods

### Subjects

A total of 44 healthy sighted individuals were recruited to participate in this study (twenty four females; average age 25.93, SD = ± 4.43). Participants have been assigned to three groups (see below). Sixteen participants were allocated to the echolocation group (ECHO—2 participants were excluded from the analysis for their inability to complete the training), 14 participants to the temporal discrimination training (TIME) and 14 to the group who did not perform any task between the 2 PPS assessment (REST). All participants reported normal touch and hearing and gave written informed consent before starting the test. The study was approved by the ethics committee of the local health service (Comitato etico, ASL 3, Genova) and conducted in line with the Declaration of Helsinki.

### Tasks and stimuli

#### Peripersonal space task

**Fig. 1:**
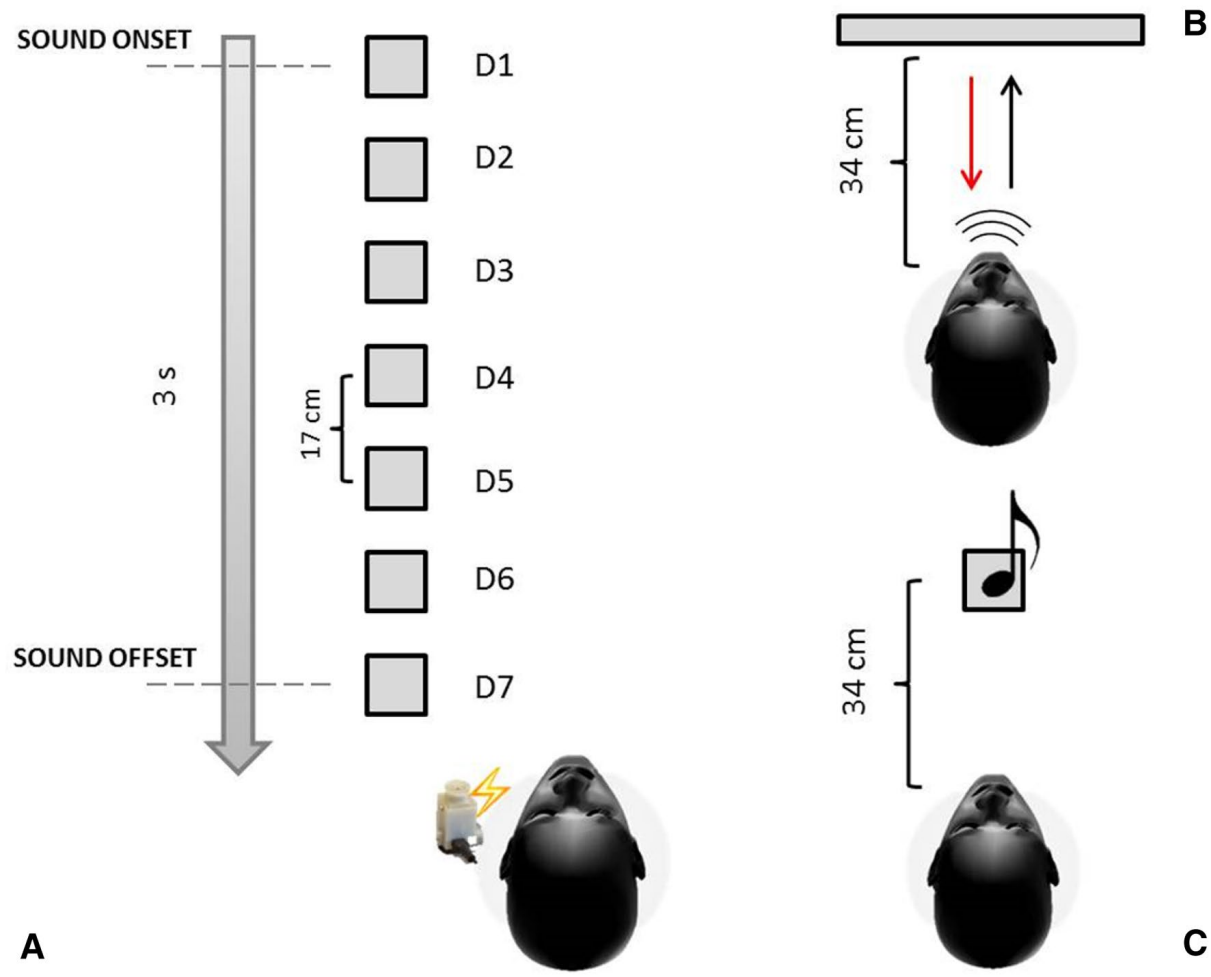
Experimental set-ups. **a** The set-up for the PPS task is shown. There were seven speakers generating sound sources at a different distance from the body. The first sound source was placed 17 cm apart from the left side of the head of each participant. The sound moved across the speakers as approaching the participant’s head (grey arrow). The vibro-tactile device was placed on the left side of the neck. The tactile stimulus was delivered when the sound was placed at one of the seven possible depicted distances (17, 34, 51, 68, 85, 102, 119). **b** The set-up for the echolocation detection task is shown. We used a bar located at 34 cm ahead the participant. The black the red arrows represent, respectively, the path of the self-generated click and the echo reflected by the bar. **c** The set-up of the temporal bisection task is shown

The task was to respond as quickly as possible to a vibro-tactile stimulation on the neck, ignoring sounds moving towards each participant. For the acoustic stimuli, we used a custom-made device comprising an array of seven serial connected loudspeakers placed on a table on the left of the participant (Fig. 1a). The first loudspeaker was located at 17 cm from the head (at the same elevation) and the last loudspeaker was at a distance of 119 cm. The distance between each loudspeaker was of 17 cm. The sounds (white noise) were originated from seven spatial sources so that we were able to precisely trigger the tactile stimulation when the sound was at the level of one of the loudspeakers located in space (Finisguerra et al. 2015). The sound moved along the distance of 102 cm in 3 s (i.e., at the speed of 34 cm/s). We sampled seven positions (17, 34, 51, 68, 85, 102, and 119 cm). For the tactile stimuli, we used a vibro-tactile custom-made device consisting of a vibration motor. The motor had a surface area of 18 mm^2^. The vibro-tactile device was placed on the left side of participants neck. Tactile stimulation lasted 20 ms. The sound and tactile stimuli were controlled through a custom-made code running on Matlab© software.

The PPS task consisted of three types of trials, randomized among the experimental block (Canzoneri et al. 2012; Serino et al. 2015b). The critical trial for the task were experimental audio-tactile trials, that were approximately 60% of the total trials. In these trials, participants heard a sound and, at a given moment in time, received a vibro-tactile stimulation, to which they were requested to respond saying ‘‘TAH’’ as quickly as possible, ignoring the auditory stimulus (Canzoneri et al. 2012). To record the time of vocal response, we performed several pilot testing in which we estimated the average noise of the experimental room (mean 52.5 dB, range 50–53 dB) and we set a threshold (53 dB) allowing to detect the 95% of the response of the participants. Then to estimate the RTs during the experiment, we subtracted the time of the vocal trigger (i.e., when the vocal response crossed the estimated threshold) from the time of the tactile stimulation. On each trial, the tactile stimulus was administered at one out of the seven temporal delays, which corresponded to a progressively shorter distance between the location of the sound and the body when the touch was given (e.g., the tactile stimulation just occurred at a specific point in time, i.e., when the sound was in a particular location in space). Approximately 20% of the trials were unimodal tactile trials, whereby the target vibro-tactile stimulus was delivered in the absence of auditory stimulation. Unimodal tactile trials were presented at two different temporal delays, before the beginning of the sound (500 ms) and after the end (500 ms) of it. These time delays have been calculated on the basis of the time that the sound takes to travel 17 cm which is the distance that separates one loudspeaker from the other. Finally, approximately 20% of trials were catch trials, through which only auditory stimuli were presented and participants were requested not to respond. These trials were included to avoid an automatic response, to assure that participants were attentive to the task and to minimize an expectancy effect intrinsic in the task (i.e., participants become faster in responding as the trial goes by as they increase their expectancy to receive the tactile target) (Kandula et al. 2017). Participants RTs were recorded by means of a microphone. Each participant performed a total of 140 trials, 28 unimodal tactile, 28 catch trials, and 12 trials for each audio-tactile combination.

Inter-trial-interval was not fixed and each trial was started by the experimenter.

#### Echolocation detection task

The task consisted in detecting an external object presented at about 34 cm from the mouth via the echo produced by emitted mouth clicks. A rectangular bar made of poly-methyl methacrylate (40 × 30 cm) was used as a target stimulus for the echolocation training. A beam microphone held the bar with the longer side placed vertically (Fig. 1b). The bar was located in front of the participant at the head level. Participants performed the task, for a total of 40 trials. The first 10 trials (practice block) were considered as practice. On the other 30 trials (training block), the percentage of correct answers was calculated (for more details see below). The bar was presented in the 50% of the trials. The participants had 20 s to give the response. To prevent from receiving any acoustic or floor vibration feedbacks due to the movement of the target, participants wore a pair of Philips SHL3000PP headphones, which played mixed music and the chair was located on a stack of rigid foam mats high 4.5 cm.

#### Auditory time bisection task

On each trial, participants heard three consecutive sounds and were requested to estimate which interval was shorter, the one between the first and the second sound or the one between the second and the third sound. The stimuli were 500 Hz tones, each having a duration of 75 ms. Sounds always came from the loudspeaker located at 34 cm from the head, i.e., at the same distance where the object for the echolocation task was placed. The experimenter took note of participants’ response at each trial. The interval duration between stimuli was determined by QUEST (Watson and Pelli 1983), an adaptive algorithm which estimates the best stimulus value to be presented after each trial, given by the current participant’s estimate. To ensure that a wide range of durations was sampled, the estimation was jittered by a random amount, drawn from a Gaussian distribution of time covering a range between 0 and 900 ms. The training included 80 trials. Inter-trial-interval was not fixed and each trial was started by the experimenter.

#### Distance perception task

Each trial was identical to the bimodal trials of the PPS, but in this case, participants were asked to verbally indicate the perceived position of the sound in space when they had felt the vibro-tactile stimulation, on a scale from 1 (very close) to 100 (very far) from the head. A total of 49 trials were performed, 7 for each position. The purpose of this task was to see whether participants were able to perceive the sound source at different locations according to their distance. It was performed by all participants.

### Procedure

All participants started the experiment blindfolded. First, we evaluated the PPS for all participants (Fig. 1a), to assess the location of their PPS boundary before any training. Then participants were divided into three groups: ECHO, TIME, and REST group. Firstly, we collected the data for the ECHO group. We decided to exclude all participants whose performance in the training block of the echolocation task was worse than their practice performance, based on the percentage of correct responses. If the percentage of correct responses in the practice was lower than the percentage at the end of the training, the participant was excluded. Then we recruited the other thirty participants for the TIME and REST group that was pseudo-randomly assigned to each group.

The first group—ECHO group—(*N* = 14; 7 females) performed an echolocation detection task after the evaluation of the PPS (see above for the task description). They were asked to sit in a different location from where the PPS task was performed so that the loudspeakers did not interfere with the echolocation task. During the change of location, participants were allowed to remove the blindfold, but they did not see the object used for the task because it was hidden by view behind a cloth. Participants had no knowledge of echolocation technique, therefore, before the beginning of the task, they received instructions on how to produce the echolocation signals with their mouth, after that they were blindfolded again. The echolocation sound was naturally produced, using no external device. While the experimenter moved the target, participants wore headphone played mixed music. Once the experimenter placed the target, the participant received a patch on the shoulder as a signal to remove the headphones and start the trial.

Participants had to judge whether the bar was in front of them or not, producing mouth clicks and estimating their echoes (Fig. 1b). All participants performed 40 trials divided into 2 blocks. During the practice block, participants received a feedback on their responses.

The second group—TIME group—(*N* = 14, 7 female) performed an auditory time bisection task as training. To maintain the same procedure as for the ECHO group, the participants of the TIME group were allowed to remove the blindfold for a few minutes, but they put it on again before beginning the time bisection task.

The third group—REST group—(*N* = 14, 8 females) simply had 30 min of break. They were allowed to remove the blindfold for just a couple of minutes. For the rest of the time, they kept the blindfold on.

All participants performed a second time the PPS task, to measure the changes in their PPS representation, and removed the blindfold at the end of the task.

Moreover, all participants performed a distance perception task to confirm that they were able to discriminate the different sound locations accordingly to the seven actual sound source positions (Canzoneri et al. 2012; Finisguerra et al. 2015).

## Results

First, we checked for any possible outliers in the RTs. We considered as outliers all the RTs below or above 2 standard deviations respect to the mean. An average of 2.34% (± 0.49) trials for the PRE condition and of 2.84% (± 0.41) for the POST condition have been excluded.

To eliminate the possibility that any effect was due to an expectancy effect, we corrected the bimodal RTs for the unimodal tactile RTs. To this aim, first we calculated the average of the tactile unimodal RTs collected before the onset of the sound in both experimental session (PRE and POST training); second, we selected the fastest RTs average between the one obtain in the PRE and POST training session; third we subtracted from the average of the raw bimodal RTs of both the experimental sessions the fastest unimodal RTs previously selected. We have made this correction for all the participant for each session.

To test whether there was a facilitation of bimodal RTs, we compared them for all the position in both session for each group against zero (that represent the facilitation threshold) using *t* test tests (with a Bonferroni correction for multiple comparisons). For the ECHO group the significant positions were 17 (*t*_14_ = − 2.1, *p* = 0.05), 51 (*t*_14_ = − 3.34, *p* < 0.05), 68 (*t*_14_ = − 2.39, *p* = < 0.05) before the training (PRE); after the training (POST) the significant positions were 17 (*t*_14_ = − 7.27, *p* < 0.01), 51 (*t*_14_ = − 7.9, *p* < 0.01), 68 (*t*_14_ = − 6.57, *p* = < 0.01), 85 (*t*_14_ = − 6.05, *p* < 0.01), 102 (*t*_14_ = − 6.39, *p* < 0.01). For the REST group the significant position both before and after the training were 17 (PRE, *t*_14_ = − 2.59, *p* < 0.05; POST, *t*_14_ = − 2.47, *p* < 0.05), 51 (PRE, *t*_14_ = − 2.35, *p* < 0.05; POST, *t*_14_ = − 2.94, *p* < 0.05). Instead for the TIME group the significant position before the training (PRE) were 17 (*t*_14_ = − 4.97, *p* < 0.01), 51 (*t*_14_ = − 2.67, *p* < 0.05), 68 (*t*_14_ = − 2.21, *p* = < 0.01), but just the first two positions after the training (POST, 17—*t*_14_ = − 3.05, *p* < 0.05 and 54—*t*_14_ = − 2.66, *p* < 0.05). Results demonstrated that in all group there was a difference in RTs between near and far positions respect to the body.

We run a three-way ANOVA with between factor GROUP (ECHO vs REST vs TIME) and two within factors Sound distance (17, 34, 51, 68, 85, 102 and 119) and Session (PRE, POST). There was a significant main effect of Session (*F*_1,39_ = 3.38, *p* = 0.05, *η*^2^ = 0.01) and Distance (*F*_6,234_ = 32.22, *p* = 3.06 × 10^−28^, *η*^2^ = 0.12). It was significant just the interaction between Group and Session (*F*_2,39_ = 5.1 *p* = 0.01, *η*^2^ = 0.04).

**Fig. 2:**
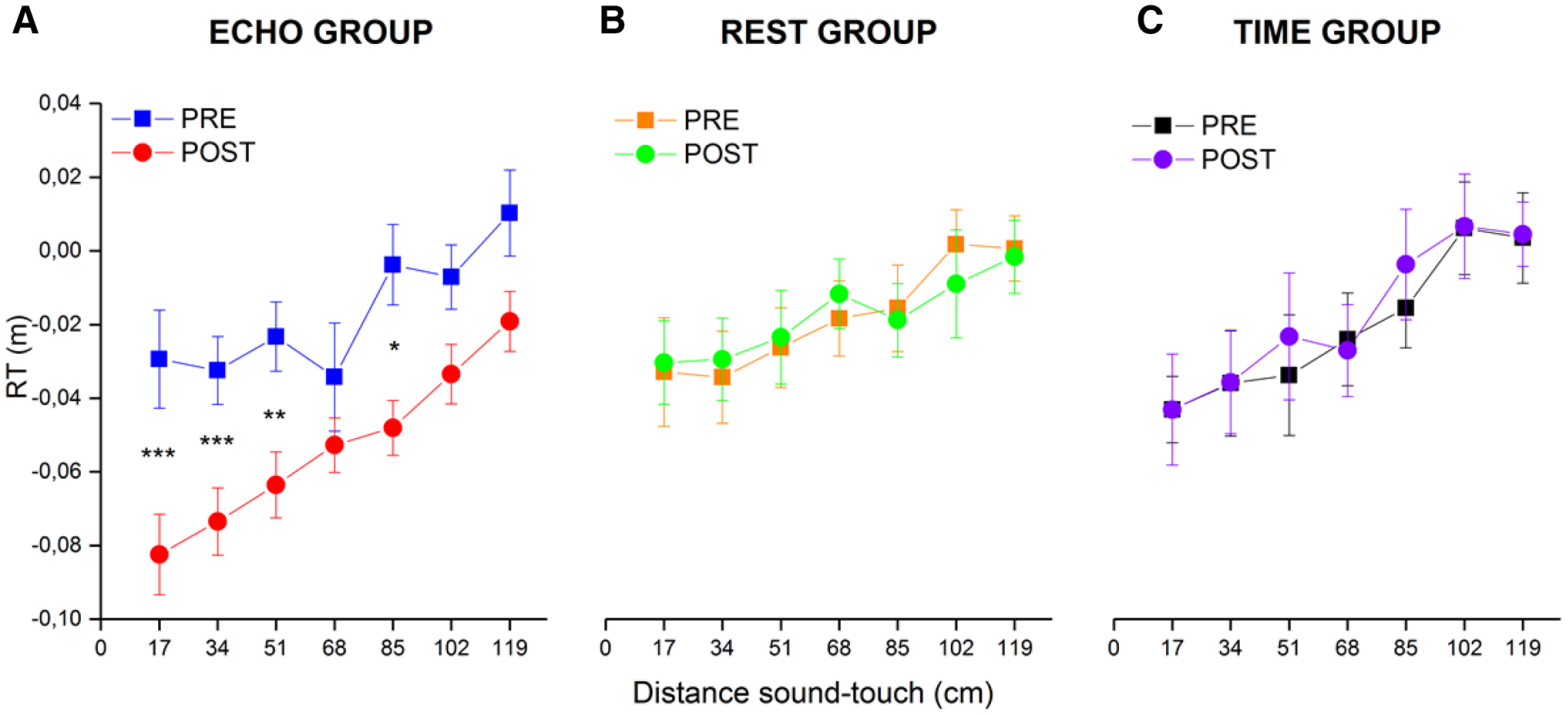
The averaged bimodal RTs (normalized for the unimodal RTs) for each group is shown as a function of the seven distances sampled during the PPS task. Data for the ECHO group before (in blue) and after (in red) the echolocation training. Data for the TIME group before (in green) and after (in orange) the time bisection task. Data for the REST group before (in magenta) and after (in cyan) 15 min of break. *Significant difference with *p* < 0.05. ***Significant difference with *p* < 0.001. The error bars represent the standard error

After, we performed three-separated ANOVAs for the baseline-corrected-RTs, one per group (Fig. 2) with the within factors of Sound distance (17, 34, 51, 68, 85, 102 and 119) and Session (PRE, POST). As expected, the main effect of distance was significant for all the groups (ECHO, *F*_6,78_ = 11.63, *p* = 2.87 × 10^−9^, *η*^2^ = 0.18; TIME, *F*_6,78_ = 13.35, *p* = 2.37 × 10^−10^, *η*^2^ = 0.12; REST, *F*_6,78_ = 8.62, *p* = 3.35 × 10^−7^, *η*^2^ = 0.07). Instead, the main effect for Session was significant only for the group ECHO (*F*_1,13_ = 25.83, *p* < 0.01, *η*^2^ = 0.19). Also the two-way interaction Sound distance × Session was significant for the Group ECHO (*F*_6,78_ = 2.28, *p* = 0.04, *η*^2^ = 0.02), and not for the other two groups (Group TIME, *F*_6,78_ = 0.06, *p* = 0.8, *η*^2^ = 0.0009; Group REST, *F*_6,84_ = 0.0001, *p* = 0.913, *η*^2^ < 0.004).

**Fig. 3:**
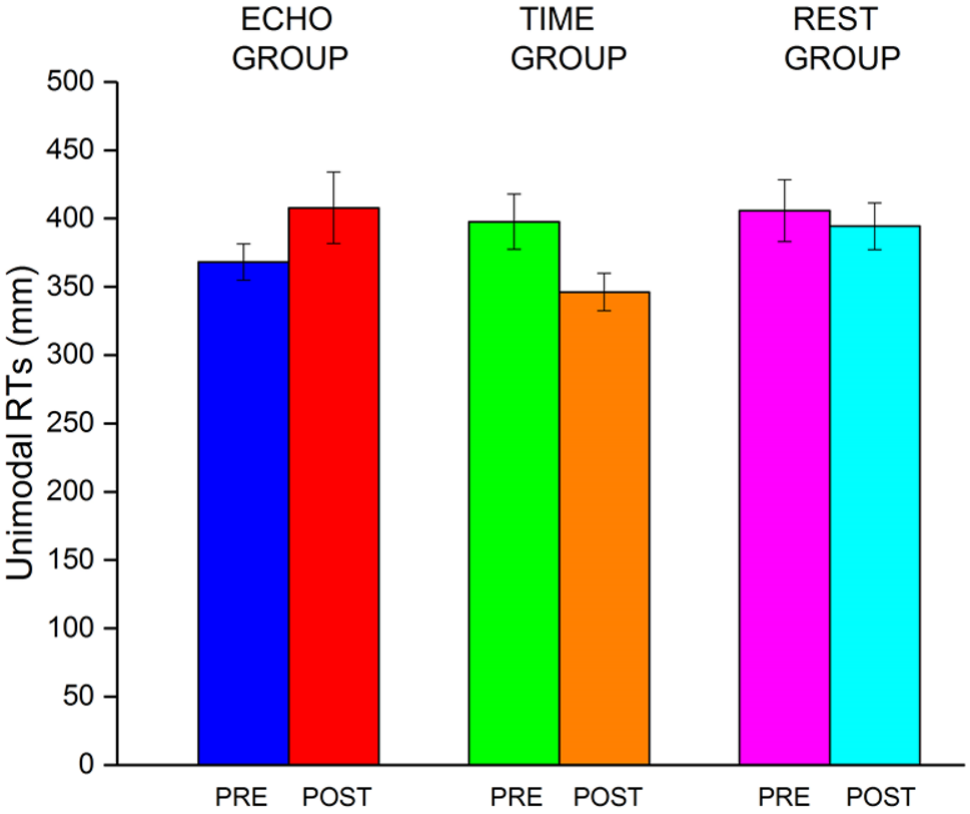
Bar plot represent the average RTs in the unimodal condition for each group before and after the auditory tasks in the Echo and Time group and for the first and second repetition in the Rest group. The error bars represent the standard error

Post hoc tests (with a Bonferroni correction for multiple comparisons) on the group ECHO (Fig. 2a) revealed a significant reduction of corrected-RTs between POST and PRE sessions for sound sources at 17 (*t*_14_ = − 5.56, *p* < 0.001), 34 (*t*_14_ = − 6.85, *p* < 0.001) and 51 (*t*_14_ = − 7.46, *p* < 0.001), 85 (*t*_14_ = − 3.48, *p* < 0.05). To control that the effect was not due to a difference on the unimodal RTs (Fig. 3), used to correct the bimodal RTs, we run a two-way ANOVA on the unimodal RTs, with the within factor Session (PRE, POST) and the between factor Group (ECHO, REST and TIME). Results showed no significant effect for either of the main factors (Session, *F*_1,39_ = 2.9, *p* = 0.1; Group, *F*_2,39_ = 2.08, *p* = 0.14), nor for the interaction (*F*_2,39_ = 1.45, *p* = 0.24). The same analysis was run using raw RTs, for more information see supplementary materials.

Finally, to control for possible differences between groups before the training, we conducted a two-way ANOVA on the bimodal RTs obtained at the first PPS session, with the factors Group (ECHO, REST and TIME) and Sound distance. The main effect of distance was significant (*F*_6,234_ = 12.39, *p* = 4.12 × 10^−12^, *η*^2^ = 0.1), whereas no main effect of Group (*F*_2,39_ = 0.03, *p* = 0.9), nor a Sound Distance × Group interaction was found (*F*_12,234_ = 1.19, *p* = 0.3).

**Fig. 4:**
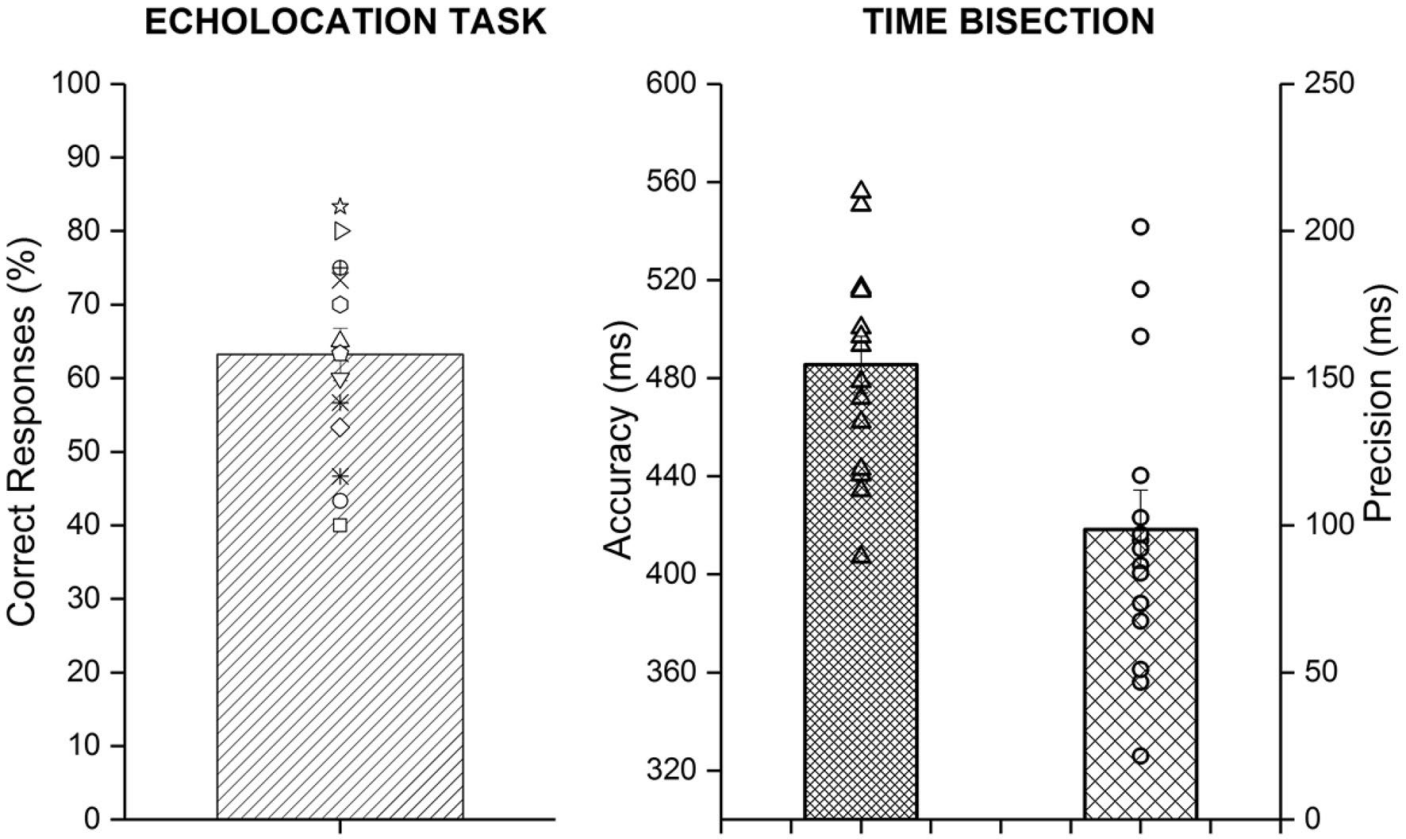
The bar plot on the left reports the average percentage of correct responses in the detection echolocation task and each empty symbols represent the result of a single participant. The plots on the right report the average of the precision and accuracy obtained in the time bisection task. The scatter plots represent the value for each participant. The error bars represent the standard error

Considering performance in the secondary tasks, we checked whether the percentage of correct responses in the detection echolocation task was better than chance level (i.e., 50%—bar plot in Fig. 4 on the left) and which was the average accuracy and precision in the time bisection (psychometric function in Fig. 4 on the right). The percentage of correct responses in the echolocation task was 60.71% (*t* test, *t*_14_ = 3.86, *p* < 0.01), significantly above the chance level. In the time bisection, in order that the second interval was judged longer than the first, it had to last, on average, 497.57 ms, with an SD of 116.25 ms (maximum duration of the interval could be 900 ms).

**Fig. 5:**
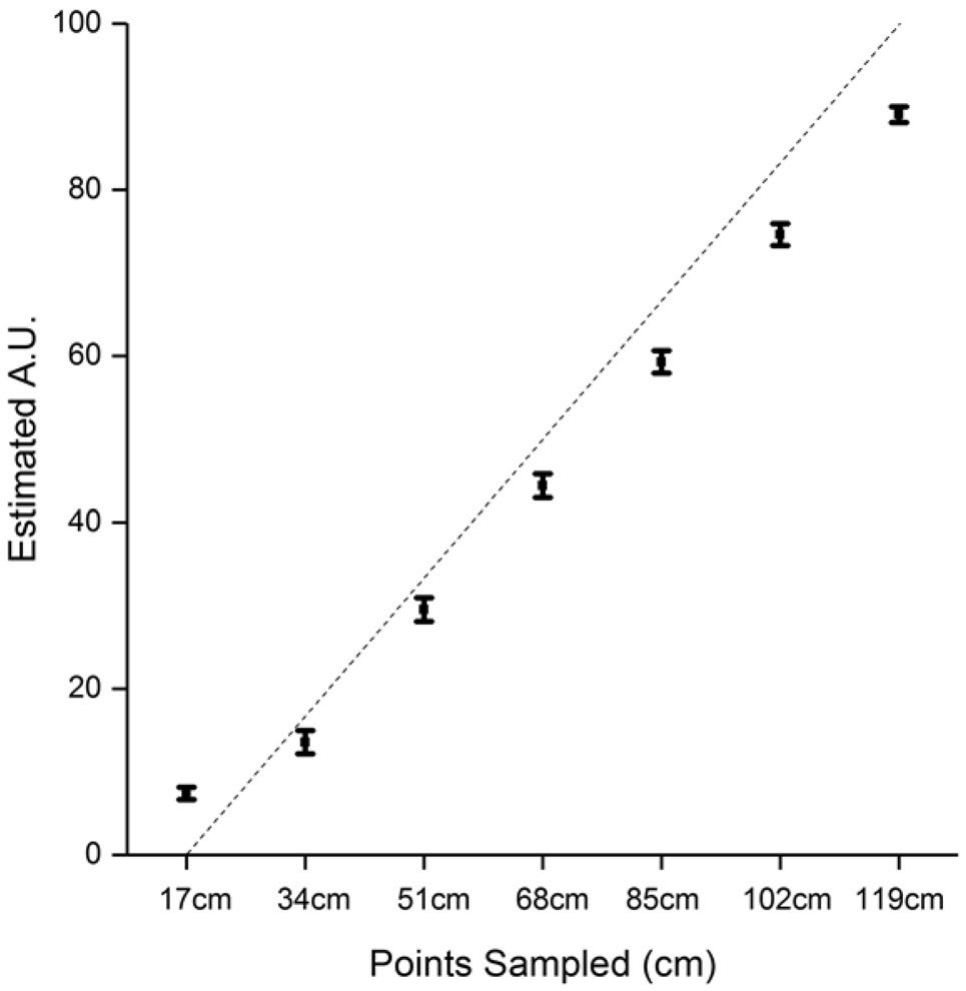
Estimate of sound distance in a scale from 1 to 100 as a function of point in space sampled for the seven-speaker set-up. Participants estimated sound distance for sounds originating from 119 cm in front (positive *x* value) and terminating at 17 cm at the head level. *y*-axis represents the average perceived value of the position of the sound on a scale from 1 to 100. The dashed line shows the equality line. The error bars represents the standard error

Finally, we checked that participants did perceive the different sounds as coming from separate locations, using the distance perception task. A repeated measure ANOVA run on participants’ responses, with Distance as a within-subject factor, indicated a main effect of Distance (*F*_6,234_ = 305.61, *p* = 1.14 × 10^−112^), indicating that, as expected, participants perceived sounds progressively closer to their body, as sounds approached. Data confirmed that participants perceived the sound source at different locations according to their distance (Fig. 5).

## Discussion

In the present study, we showed that performing echolocation training with stimuli in the near space, affected multisensory interaction within the PPS. The training consists in detecting an external object presented at about 34 cm from the body via the echoes produced by the echolocation signals (mouth clicks). Compared to the RTs before the training, the RTs to tactile stimuli, coupled with looming sounds, speeded up around the area where the echolocation training was conducted.

This effect might not depend on a learning process of the task used to evaluate the PPS, due to task repetition. Indeed, participants of the REST group, who were tested twice after the same amount of time as for the ECHO group, did not show any change in their RTs between the first and the second PPS assessment.

Another explanation for the present effect is that the change in the multisensory interaction of the ECHO group within the PPS might be due to a general enhanced attention towards auditory stimuli in the near space. However, a shift of attention cannot explain, per se, the results of the ECHO group. Indeed, the participants of TIME group—who were engaged in a demanding task on auditory stimuli occurring exactly at the same location as the echolocation training—did not show any specific changes in multisensory interaction after the training.

A difference between the TIME group and the ECHO group is in the nature of the task performed in the near space. In the former is required to analyze temporal intervals between the sounds. On the contrary, the echolocation task is focused on acquire spatial feature from auditory cues. It can be argued that time bisection task can involve spatial component, in line with the view that temporal intervals are also mentally represented accordingly to spatial representations (Bonato et al. 2012). However, there are two fundamental differences between the mental processes involved in the TIME and ECHO tasks. Firstly, time–space overlap in mental representation acts on the lateral dimension of space (Vallesi et al. 2008), whereas the echolocation task, and the related changes in PPS representation, occur in depth. Secondly, the temporal training implied a pure perceptual task related to stimuli presented in a given position of space, whereas the echolocation task also implied a sensory-motor component: participants performed an action (i.e., emitting a sound with their mouth) and processed the sensory consequences of that action (i.e., the echo produced) in space. We suggest that this second sensory-motor aspect of the task is critical to determine its effect on multisensory processing. The sensory-motor spatial task implied in the echolocation training resembles tool-use. The action with a tool allows people to extend their action possibilities in order to get sensory information from the far space. Previous studies showed that PPS can be modified by tool-use training (Maravita and Iriki 2004; Holmes et al. 2007; Canzoneri et al. 2013b; Martel et al. 2016). Conversely, displace spatial attention towards the farther space to point towards far objects (Canzoneri et al. 2013b) or passively hold a tool (Farnè and Làdavas 2000) are not sufficient actions for PPS extension. More recently, Serino et al. (2015a) proposed a neural network model to explain plasticity in PPS representation induced by tool-use via multisensory congruency. They showed that the temporal congruency between a stimulus on the body and an auditory feedback from the far space drives the extension of multisensory integration towards the location of the sensory feedback. A similar mechanism can be suggested to explain the effect of the echolocation training: participants, producing the clicks with their mouth, performed a movement that generates a time coherent feedback between the tactile stimulation (mouth) and the echo from a further spatial location during the training. Our results suggest that the repetition for a given amount of time of such activity induces a specific effect on PPS processing for space where the training is performed.

In the present protocol, the object in the echolocation training was placed in a fixed position, near the participant. The present findings showed that, actually, the training did not induce an extension of the participants’ PPS, rather, it increased multisensory interaction around the location of the object (with a possible weaker effect for the origin of the echo generated by the room walls). The increase of multisensory processing in the near space might be seen as a difference compared to previous reports about the classic extension of PPS induced by tool-use. The present results do not show any PPS extension. However, previous tool-use studies (Farne et al. 2005; Farnè et al. 2005, 2007) also showed that the change of PPS processing is specific to the location where the tool is functionally used. Therefore, it occurs at closer distances from the body at the level of the functional part of the tool (Ursino et al. 2007; Magosso et al. 2010; Gallivan et al. 2013).

Echolocation is mainly used by blind people to locate objects in space or to navigate through the environment, to avoid obstacles. A similar function is achieved by blind people using the white cane. Interestingly, Serino et al. (2007) showed that a short training with a white cane is sufficient to temporarily modify PPS representation in sighted participants, whereas long-term blind cane users show a PPS representation which is extended toward the tip of the cane, as if the cane constitutes the new boundary of their PPS (Witt et al. 2005). Such a remapping of PPS representation seems to have an adaptive value, allowing to locate in advance a possible harmful object before it collides with the body (Rossetti et al. 2015). Unlike the white cane, that physically allows reaching the far space, echolocation allows the blind person to detect objects thanks to the interpretation of the echoes produced by the reflections of sounds. Therefore, we propose that echolocation is a way to reduce the lack of information about the space between the body and an external object in absence of visual cues. Increasing multisensory processing for that portion of space might be a key mechanism to achieve this function. Further investigations are needed to test whether echolocation can be compared to tool-use in the far space producing an extension of PPS.

Another point to highlight is related to how the task of the PPS is designed. It might occur that the participants react to tactile stimulation not using spatial cues. Indeed, it is possible that when the tactile stimulation occurs the reaction is based on the time delay from the begging of the sound and the tactile stimulation or on the duration of the sound. Therefore, it might be that the effect of echolocation training is not specific for space, but for time.

The issue about the temporal or spatial nature of the PPS task has been address by several experimental studies in the past. Canzoneri et al. (2012) compared the RTs between looming and receding sounds with tactile stimuli, given at the same delay, and associated to complementary and specular distances. The authors reported that the speeding up of tactile RTs depended on the distance of the sound and not by the temporal delay between the beginning of the sound and the tactile stimulation. Although the effect was stronger for looming sounds, as expected by the response properties of PPS neurons (Graziano and Cooke 2006). Similar findings have been replicated by Serino et al. (2015b); see also Salomon et al. (2017) for the visual version of the task with subthreshold stimuli). More recently, Finisguerra et al. (2015) showed a space-dependent and a delay-independent increase of motor evoked potentials induced by TMS stimulation over the primary motor cortex for both looming and receding sounds. Moreover, Noel et al. (2018) found a spatially dependent facilitation effect on RTs as a function of the velocity of the looming stimuli, i.e., faster sounds are associated with an extended PPS boundary. These results are in line with neurophysiology data on monkeys showing that the receptive fields of PPS neurons are more extended when probed with faster visual stimuli compared to slower ones (Fogassi et al. 1996).

Based on evidence in the literature, we can state that the enhancing of RTs is due to the spatial position of the sounds in space, even if an expectancy effect due to the temporal modulation of the task does exist (Kandula et al. 2017; Pellencin et al. 2018). Such effect is significantly weaker than the effect due to the position in space and it has been repetitively dissociated from it.

Moreover, if the task of PPS is based on time delay, why it has been found an effect just on the ECHO group and not in the TIME group, in which the training was specific for time intervals? This brings us back to the explanation, we gave above. We would like to point out that echolocation is not a technique that uses only spatial information. Echolocation use the delay between the sound produced and the coming back echoes to infer spatial information. So in both cases, PPS task and echolocation, there is a temporal and a spatial component, but the main component remains space.

To conclude, in this work we have shown—for the first time, to the best of our knowledge—that the representation of the PPS around the head can be modified by echolocation. This effect is not related to a training effect, nor to focusing attention to a specific spatial location. It likely depends on the plastic property of the PPS system, which adapts as a function of the congruency between a body action and a sensory feedback from a given position in space.

## Electronic supplementary material

Below is the link to the electronic supplementary material.

Supplementary material 1 (DOCX 138 KB)
