# Supplementary material for: Enhanced audio-tactile multisensory interaction in a peripersonal task after echolocation
Source: Exp Brain Res. 2019 Jan 7;237(3):855–64. doi: 10.1007/s00221-019-05469-3 (PMC6394550; doi:10.1007/s00221-019-05469-3)
Supplement: Supplementary file 1 — Supplementary material 1 (DOCX 138 KB) [file 221_2019_5469_MOESM1_ESM.docx]

Supplementary materials.

Supplementary Results.

To see whether the effect found in the main analysis was due to a specific effect of the echolocation training and not to the unimodal correction applied to the bimodal RTs, we run the same kind of analysis using raw RTs.

After, we performed three separated ANOVAs one per group (see figure 1) with within factors of Sound distance (17, 34, 51, 68, 85, 102 and 119) and Session (PRE, POST). As expected, the main effect of distance was significant for all the groups (ECHO, F_6,78_ = 11.63, p = 2.88*10^-9^, ɳ^2^ = 0.08; TIME, F_6,78_ = 13.35, p = 2.37*10^-10^, ɳ^2^ = 0.03; REST, F_6,78_ = 8.62, p = 3.35*10^-7^, ɳ^2^ = 0.03). Instead, the main effect for Session was significant only for the group ECHO (F_1,13_ = 25.82, p < 0.01, ɳ^2^ = 0.08). Also the two-way interaction Sound distance × Session was significant for the Group ECHO (F_6,78_ = 2.28, p = 0.04, ɳ^2^ = 0.008), and not for the other two groups (Group TIME, F_6,78_ = 0.06, p = 0.8, ɳ^2^ = 0.00019; Group REST, F_6,84_ = 0.0001, p = 0.913, ɳ^2^ = 0.001).

Post hoc tests (with a Bonferroni correction for multiple comparisons) on the group ECHO (figure 1) revealed a significant reduction of RTs between POST and PRE sections for sound sources at 17 (t_14_ = -5.56, p < 0.001), 34 (t_14_ = -6.85, p < 0.001) and 51 (t_14_ = -7.46, p < 0.001), 85 (t_14_ = -3.48, p < 0.05).


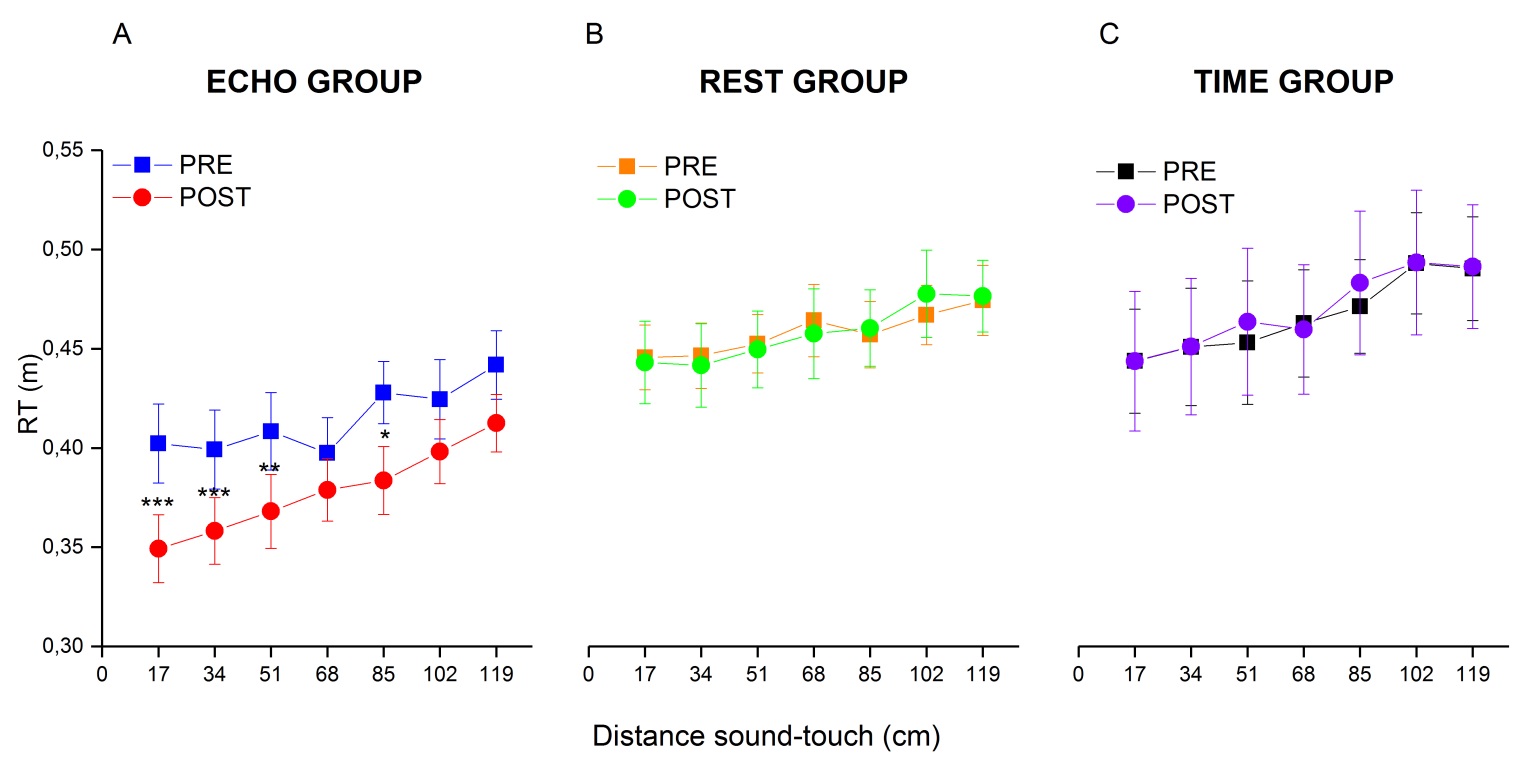


**Figure 1**. The averaged bimodal RTs (normalized for the unimodal RTs) for each group is shown as a function of the seven distances sampled during the PPS task. Data for the ECHO group before (in blue) and after (in red) the echolocation training. Data for the TIME group before (in green) and after (in orange) the time bisection task. Data for the REST group before (in magenta) and after (in cyan) 15 minutes of break. (*) indicates a significant difference with p < 0.05. (***) indicates a significant difference with p < 0.001. The error bars represent the standard error.
